# Cognitive status and use of analgesics and anxiolytics in residents of nursing homes in the Czech Republic

**DOI:** 10.2147/CIA.S188601

**Published:** 2018-12-11

**Authors:** Iva Holmerová, Stefanie R Auer, Anna Beránková, Margit Höfler, Paulina Ratajczak, Michal Šteffl

**Affiliations:** 1 Centre of Expertise in Longevity and Long-Term Care, Faculty of Humanities, Charles University, Prague, Czech Republic; 2 Danube University Krems, 3500 Krems, Austria; 3 MAS Alzheimerhilfe, 4820 Bad Ischl, Austria; 4 Faculty of Physical Education and Sport, Charles University, Prague, Czech Republic, steffl@ftvs.cuni.cz

**Keywords:** pain, anxiety, dementia, analgesics, anxiolytics

## Abstract

**Background:**

The treatment of pain and treatment of anxiety are two of the most complex issues in nursing homes worldwide, mainly because of the large numbers of people with cognitive impairment who reside in this environment.

**Aim:**

The aim of this study was to investigate the administration of analgesics and anxiolytics to people living in nursing homes, taking into account cognitive status.

**Methods:**

For this cross-sectional study, we used data from 404 residents who lived in nine randomly selected nursing homes in the Czech Republic and participated in the Czech-Austrian Long-Term Care Research Project called DEMDATA. Information about medication administration was obtained from medical records. Cognitive impairment was evaluated by the Mini-Mental State Examination, and pain was measured by the Pain Assessment in Advanced Dementia. To measure anxiety, we used the Behavioral Pathology in Alzheimer’s Disease Rating Scale in residents with severe-to-moderate dementia and also the standardized questionnaire EuroQol in other residents.

**Results:**

In all, 68% (95% CI 62–74) of residents with pain did not receive any analgesic medication and 31% (95% CI 25–38) of residents were administered some analgesics and continued to report pain. The lowest reported occurrence of pain, as well as the lowest prevalence of analgesic administration, was in residents with moderate-to-severe dementia. We found that an alarming percentage of residents in the nursing homes were not treated sufficiently.

**Conclusion:**

This study indicates that treatment effects should be better monitored.

## Introduction

Dementia, anxiety, and depression are the most common psychiatric disorders in nursing homes.^1^ These conditions, together with pain, may decrease the quality of life of older persons. Maintaining quality of life is a challenge for caregivers and clinicians in nursing homes because many nursing home residents are older than 85 years and have at least one of these conditions. More than two-thirds of care home residents are described as having dementia,^2^ while anxiety symptoms were found in 6.5%–58.4%^3^ and pain prevalence varied from 60% to 70%.^4^

It is relatively well known that cognitively impaired nursing home residents are prescribed and administered significantly less analgesic medications, in terms of both frequency and dosage, than their more cognitively intact peers.^5^ Why those with more severe cognitive impairment receive fewer analgesics than others is not quite clear.^6^ Nevertheless, even when a person with dementia is recognized as being in pain, the prescription of analgesic medications often falls short of best-practice recommendations.^7^ While analgesic administration in people with dementia is known to be inadequate, the use of anxiolytics has been found to be unnecessarily high among nursing home residents.^8^ However, the use of anxiolytics, especially benzodiazepines, in the older population and for people with dementia, is considered to be inappropriate, and consequently, their administration is no longer recommended for this population.^9^–^11^

As far as possible, nursing home staff should make every effort to assist residents to maintain quality of life in their later years, which includes the opportunity to live without pain and anxiety. In this study, we focused on the administration of analgesics and anxiolytics by specifically exploring the presence of symptoms for which they were indicated and also the relationship between the administration of these medicines and the level of cognitive impairment.

## Methods

### Study population

A total of 14 nursing homes in the Czech Republic were randomly selected to participate in the DEMDATA study. Seven of the 14 nursing homes were publicly owned (municipal), five were run by a non-profit organization, and two were run by a for-profit organization. The homes were situated in Prague (Czech capital) and Central Bohemia.^12^ All people living in the nursing homes had the same opportunity to participate, although those with an acute health condition (ie, intensive care), or patients who were dying, were excluded. In all, 964 residents were invited to take part in the study and 514 residents agreed to participate. Medical records were not available in five participating homes due to their policies concerning data safety. Finally, data from 404 residents were used in this study.

Participants signed an informed consent form. In the case where written consent could not be given, a legal representative or a relative was required to provide proxy consent. The study protocol and study methodology were approved by the ethics committee of the Center of Gerontology in Prague, Czech Republic. All the assessments were carried out by trained assistants who had undertaken higher education training in the health and/or social care field and were specially trained to carry out the assessments. This study was conducted in accordance with the Declaration of Helsinki.

### Cognitive impairment

Cognitive impairment was assessed using the Mini-Mental State Examination (MMSE).^13^ The score of the MMSE ranges from 0 to 30 points, and a higher score indicates better cognitive functioning. In this study, 0–17 points represented severe-to-moderate dementia, 18–23 represented mild dementia, and 24–30 represented no cognitive impairment.

### Pain and anxiety

In residents with severe-to-moderate dementia according to the MMSE, we used the Pain Assessment in Advanced Dementia (PAINAD)^14^ for pain detection and parts E and G from the Behavioral Pathology in Alzheimer’s Disease Rating Scale (BEHAVE-AD)^15^ for identifying anxiety. They both are sufficiently sensitive, simple, valid, and reliable instruments for use with people with cognitive impairment. In residents with mild dementia and with no cognitive impairment according to the MMSE, we used the EuroQol^16^ to detect pain and anxiety. We used the EQ-5D-3L version with three responses: a resident had no/mild/severe pain and a resident was not/mildly/severely anxious. For the purposes of the analyses, we then categorized patients into two categories: those who had no pain or anxiety and those who had mild-to-severe pain or anxiety.

### Medication

Information about medication was obtained from medical records and verified with care staff. For the purposes of this study, we only recorded medications by their active substances and we did not record any information about their exact daily dosages. We categorized medication according to their active substances as the following: mild analgesics (metamizole, paracetamol/acetaminophen), non-steroidal anti-inflammatory drugs (diclofenac, ibuprofen, meloxicam, nimesulide, tiaprofenic acid), opiates (tramadol, buprenorphine, codeine, dihydrocodeine, fentanyl, morphine, hydromorphone, oxycodone), and anxiolytics-benzodiazepines (alprazolam, bromazepam, clonazepam, midazolam, oxazepam).

### Statistical analyses

In descriptive statistics, mean and SD values were calculated for age and percentages were calculated for all the other variables. To test for differences among the groups according to cognitive impairment, we used the Kruskal–Wallis test for age and the Pearson chi-squared test for the other variables in the descriptive statistics. A contingency table 2×2 was created to show a proportion of people who had/did not have a condition and were given/not given drugs. In these contingency tables, we calculated percentages and 95% CI. Statistics were calculated using IBM SPSS Statistics 23.

## Results

The average age of participants was 84.8±7.5 years, and 78.0% were female. According to the MMSE scores, 63.4% had dementia. 50.7% experienced pain, and 32.4% experienced anxiety. Except for age, gender, and anxiety, all other variables differed significantly among the groups that were divided according to cognitive impairment. The lowest percentage of pain, as well as the lowest percentage of analgesic administration, was among residents with severe-to-moderate dementia. Descriptive statistics are presented in Table 1.

Among all the residents with pain (n=205), 68% (95% CI 62–74) did not receive any analgesic medications. The highest percentage 87% (95% CI 76–93) was in residents with severe-to-moderate cognitive impairment (n=67). However, 31% (95% CI 25–38) of residents were given some analgesics and still reported pain. A relatively low number of residents were administered anxiolytics. Some residents, (n=92) 36% (95% CI 23–51), with no cognitive impairment who reported anxiety received anxiolytics and still reported anxiety. Many of the other cognitively impaired residents who experienced anxiety did not receive any anxiolytics. Out of all the participants, the highest percentage was among those with severe dementia where, according to the BEHAVE-AD, 60 residents had anxiety and 91% (95% CI 81–96) did not receive any anxiolytics. Medication intakes according to the presence of conditions among different groups are shown in Table 2.

## Discussion

In this study, our main finding was that almost 70% of residents were not administered any painkillers even though they experienced pain. This statistic is alarming and is also higher than results from a previously published study where the percentage was 62.5%^17^ and much higher than another study of 1,387,405 long-stay residents in the US where of residents in persistent pain, 6.4% were untreated and 32.0% were undertreated.^18^ Furthermore, it is substantially higher than a study from the previous wave of the linked minimum data set (MDS), where of 18,526 residents with persistent pain, 3,094 (16.7%) did not receive prescription analgesics.^19^

We also found that, in general, many residents who were prescribed analgesics continued to report pain. This is a clear indication that the effect of analgesics was not appropriately monitored; possibly doses were not sufficient, which may explain why residents continued to report pain.

Our findings support the idea that pain is less recognized in people with dementia. However, the communication of pain presence, which is not obvious and is not understood by caregivers, is a well-known problem in dementia care.^20^ Different authors have reported that the prevalence of pain in dementia patients is less frequent compared to that in age-matched subjects without cognitive impairment.^21^ We also found huge differences in analgesic use when residents were divided according to cognitive impairment. Participants with cognitive impairment were given lower amounts of all types of analgesics compared to participants without cognitive impairment. This fact has been described previously by a number of authors,^5^,^6^,^22^,^23^ and our data confirmed that this may also be occurring in the Czech Republic.

The overall percentage of residents with anxiety was lower when compared to those with pain. We found almost the same prevalence of anxiety as has been published previously.^24^,^25^ Our results showed higher levels of anxiety among residents with severe-to-moderate dementia than in both residents without cognitive decline and those with mild dementia, but the difference was not statistically significant. The prevalence of anxiety may vary among dementia subtypes,^26^ but we did not focus on different types of dementia in this study.

Regarding the use of anxiolytics, the frequency of administration in our study was also lower among people with cognitive impairment compared to people without cognitive impairment. This can be linked to the recent developments and thorough education in the Czech Republic about the negative side effects of these drugs for people with dementia. The use of anxiolytics, and especially benzodiazepine drugs for people with dementia, is considered to be inappropriate, and these drugs are no longer used as a result of long-term educational campaigns and effective guidelines.^9^–^11^ A relatively large proportion of residents used other kinds of medication that were not considered in this study, such as antidepressants or antipsychotics, which might be used as off-label adjuvant therapy. Nevertheless, the overall percentage of residents who had anxiety and did not use any anxiolytics was very high in all the groups.

## Limitations

Several limitations of this study should be mentioned. First, the study was cross-sectional, and although we used standardized methods to obtain information about pain and anxiety, we did not focus on their possible origin. To diagnose dementia, we divided residents only according to their performance in the MMSE and were not able to consider the type of dementia. Furthermore, we did not focus on separate drugs and did not include any information about possible comorbidities. Although medication use was verified by care staff, it is possible that we were not able to obtain all information about over-the-counter (OTC) drugs. Their use is very limited, particularly in this population (nursing homes) due to financial reasons, such as high cost of accommodation and other services. Moreover, an off-label use of drugs was not considered among the aims of this study. However, this may have influenced the results.

This study has uncovered a suboptimal situation as regards to the administration of medications in nursing homes in the Czech Republic. Our primary concern resulting from this study is the insufficient treatment of pain. In order to ensure sustained quality of life for residents of nursing homes, the reasons behind this situation should be addressed as a priority.

## Figures and Tables

**Table 1:** Descriptive statistics

	All (N=404)	No cognitive impairment (n=148)	Mild dementia (n=85)	Severe-to-moderate dementia (n=171)	Significance

Age	84.8 (7.5)	83.7 (7.3)	85.6 (7.8)	85.3 (7.4)	0.849
Females	79.0	74.3	77.6	83.6	0.120
Cognitive impairment (MMSE)	63.4	36.6	21.0	42.4	<0.001
Pain	50.7	62.2	54.1	39.2	<0.001
Anxiety	32.4	28.4	34.1	35.1	0.414
Analgesics	28.2	40.5	25.9	18.7	<0.001
Anxiolytics	14.4	22.3	11.8	8.8	0.002

**Notes:** Values are expressed as mean (SD) or percentages. The difference between the groups was tested using Pearson chi-squared test for categorical variables and the Kruskal–Wallis test for age.

**Abbreviation:** MMSE, Mini-Mental State Examination.

**Table 2:** Proportions of residents according to medicament administration and presence of conditions

	All (N=404)		No cognitive impairment (n=148)		Mild dementia (n=85)		Severe-to-moderate dementia (n=171)	

Pain	**No** n=199	**Yes** n=205	**No** n=56	**Yes** n=92	**No** n=39	**Yes** n=46	**No** n=104	**Yes** n=67
Analgesics								
No	75 (68–80)	68 (62–74)	63 (49–74)	58 (47–67)	85 (70–93)	65 (51–77)	78 (69–85)	87 (76–93)
Yes	25 (20–32)	31 (25–38)	38 (26–51)	42 (33–53)	15 (7–30)	35 (23–49)	22 (15–31)	13 (7–24)
Anxiety	**No** n=273	**Yes** n=131	**No** n=106	**Yes** n=42	**No** n=56	**Yes** n=29	**No** n=111	**Yes** n=60
Anxiolytics								
No	88 (83–91)	81 (74–87)	83 (75–89)	64 (49–77)	89 (79–95)	86 (69–95)	91 (84–95)	91 (81–96)
Yes	12 (9–17)	18 (13–26)	17 (11–25)	36 (23–51)	11 (5–21)	14 (6–31)	9 (5–16)	8 (3–18)

**Note:** Values are expressed as percentages and 95% CI.
